# Increased brain age and relationships with blood-based biomarkers following concussion in younger populations

**DOI:** 10.1007/s00415-023-11931-8

**Published:** 2023-08-18

**Authors:** Andrew R. Mayer, Timothy B. Meier, Josef M. Ling, Andrew B. Dodd, Benjamin L. Brett, Cidney R. Robertson-Benta, Daniel L. Huber, Harm J. Van der Horn, Steven P. Broglio, Michael A. McCrea, Thomas McAllister

**Affiliations:** 1 The Mind Research Network/Lovelace Biomedical and Environmental Research Institute, 1101 Yale Blvd. NE, Albuquerque, NM 87106 USA; 2 Neurology and Psychiatry Departments, University of New Mexico School of Medicine, Albuquerque, NM USA; 3 Department of Psychology, University of New Mexico, Albuquerque, NM USA; 4 Department of Neurosurgery, Medical College of Wisconsin, Milwaukee, WI USA; 5 Department of Biomedical Engineering, Medical College of Wisconsin, Milwaukee, WI USA; 6 Department of Cell Biology, Neurobiology and Anatomy, Medical College of Wisconsin, Milwaukee, WI USA; 7 Department of Neurology, Medical College of Wisconsin, Milwaukee, WI USA; 8 Michigan Concussion Center, University of Michigan, Ann Arbor, MI USA; 9 Department of Psychiatry, Indiana University School of Medicine, Bloomington, IN USA

**Keywords:** Concussion, Brain age, Neural biomarkers, Inflammatory biomarkers

## Abstract

**Objective:**

Brain age is increasingly being applied to the spectrum of brain injury to define neuropathological changes in conjunction with blood-based biomarkers. However, data from the acute/sub-acute stages of concussion are lacking, especially among younger cohorts.

**Methods:**

Predicted brain age differences were independently calculated in large, prospectively recruited cohorts of pediatric concussion and matched healthy controls (total *N* = 446), as well as collegiate athletes with sport-related concussion and matched non-contact sport controls (total *N* = 184). Effects of repetitive head injury (i.e., exposure) were examined in a separate cohort of contact sport athletes (*N* = 82), as well as by quantifying concussion history through semi-structured interviews and years of contact sport participation.

**Results:**

Findings of increased brain age during acute and sub-acute concussion were independently replicated across both cohorts, with stronger evidence of recovery for pediatric (4 months) relative to concussed athletes (6 months). Mixed evidence existed for effects of repetitive head injury, as brain age was increased in contact sport athletes, but was not associated with concussion history or years of contact sport exposure. There was no difference in brain age between concussed and contact sport athletes. Total tau decreased immediately (~ 1.5 days) post-concussion relative to the non-contact group, whereas pro-inflammatory markers were increased in both concussed and contact sport athletes. Anti-inflammatory markers were inversely related to brain age, whereas markers of axonal injury (neurofilament light) exhibited a trend positive association.

**Conclusion:**

Current and previous findings collectively suggest that the chronicity of brain age differences may be mediated by age at injury (adults > children), with preliminary findings suggesting that exposure to contact sports may also increase brain age.

**Supplementary Information:**

The online version contains supplementary material available at 10.1007/s00415-023-11931-8.

## Introduction

There is increased concern that concussion (used synonymously with mild traumatic brain injury; mTBI), as well as repetitive head impact exposure (hereafter referred to as exposure effects), may result in neurobehavioral sequelae and neuropathological changes that persist for years post-injury [1]. Specifically, a variety of macrostructural changes (e.g., septum pellucidum with septal fenestrations, ventriculomegaly, reduced cortical volume) have been qualitatively observed following repetitive mTBI decades post-injury on MRI [2–5], and within the first few months to years post moderate-to-severe TBI (msTBI) [6, 7]. A growing body of evidence suggests that the acute effects of concussion can also be detected in vivo among younger samples using more quantitative imaging techniques [8, 9] as well as with blood-based [10–12] biomarkers, albeit to more subtle degrees.

The brain age gap estimation method [13] has been applied to various neuropsychiatric populations to quantify accelerated brain age [14], risk of cognitive dysfunction [15, 16], and even mortality [17]. The predicted age difference [PAD: difference between predicted age obtained from magnetic resonance imaging (MRI) data and chronological age] has excellent psychometric properties and is clinically intuitive [14, 18]. Moderate-to-severe TBI studies have reported positive relationships between time post-injury and PAD, as well as longitudinal changes in brain age and/or decreased brain volume [7, 15, 19, 20], but see [21]. These findings collectively suggest a progressive decline rather than a “one-off” decrease in brain volume post-msTBI, with one study reporting declines in both grey (+ 4.66 years) and white (+ 5.97 years) matter [15]. Blood levels of glial fibrillary acidic protein (GFAP) and neurofilament light (NFL) have also been associated with microstructural injury acutely and predict brain volume loss (NFL more than GFAP) 5 years or more post-injury following msTBI [12, 19, 20].

In contrast, longitudinal studies of putative changes in brain age following mTBI remain relatively sparse [1, 7]. Two recent studies observed increased PAD during acute mTBI that persisted 6–12 months post-injury using diffusion [22] or structural [23] MRI. Both of these studies also suggested that PAD was increased for geriatric relative to adult mTBI patients in an age-dependent gradient. However, other cross-sectional chronic mTBI studies have reported null effects [15], or only observed PAD differences in a subset of chronic (i.e., years to decades post-injury) veteran mTBI sample [24]. Few studies have investigated the effects of acute mTBI and recovery on PAD across the full spectrum of mTBI [25], including the potential of moderating effects of concussion history/repetitive head impacts on PAD in high-risk younger athletes, as well as in younger samples following acute concussion. There are several potential confounding effects of neurodevelopment (e.g., cortical thinning during adolescence) on brain age estimation that require consideration in younger samples.

The current study therefore examined acute/sub-acute (i.e., first week of injury) and early chronic (4–6 months) effects of concussion on PAD in two large independent cohorts of concussed pediatric emergency room patients and collegiate athletes. The inclusion of multiple groups permitted for replication of acute findings as well as examination of putative exposure effects to repetitive head injury (athlete sample only). Brain age was calculated using an algorithm that was developed on a large sample that spanned the developmental continuum to maximize generalizability [26]. A priori hypotheses were that PAD would be increased during the acute and sub-acute phases of concussion [22, 23], as well as increased secondary to exposure effects and past history of concussions. We further hypothesized [12, 19, 20] that blood-based measures of axonal injury and astrogliosis (NFL, GFAP, total tau) would be more strongly associated with increased PAD relative to inflammatory markers [interleukins (IL) 6 and 10, tumor necrosis factor-alpha (TNF-α)].

## Methods

### Pediatric cohorts

**Fig. 1:**
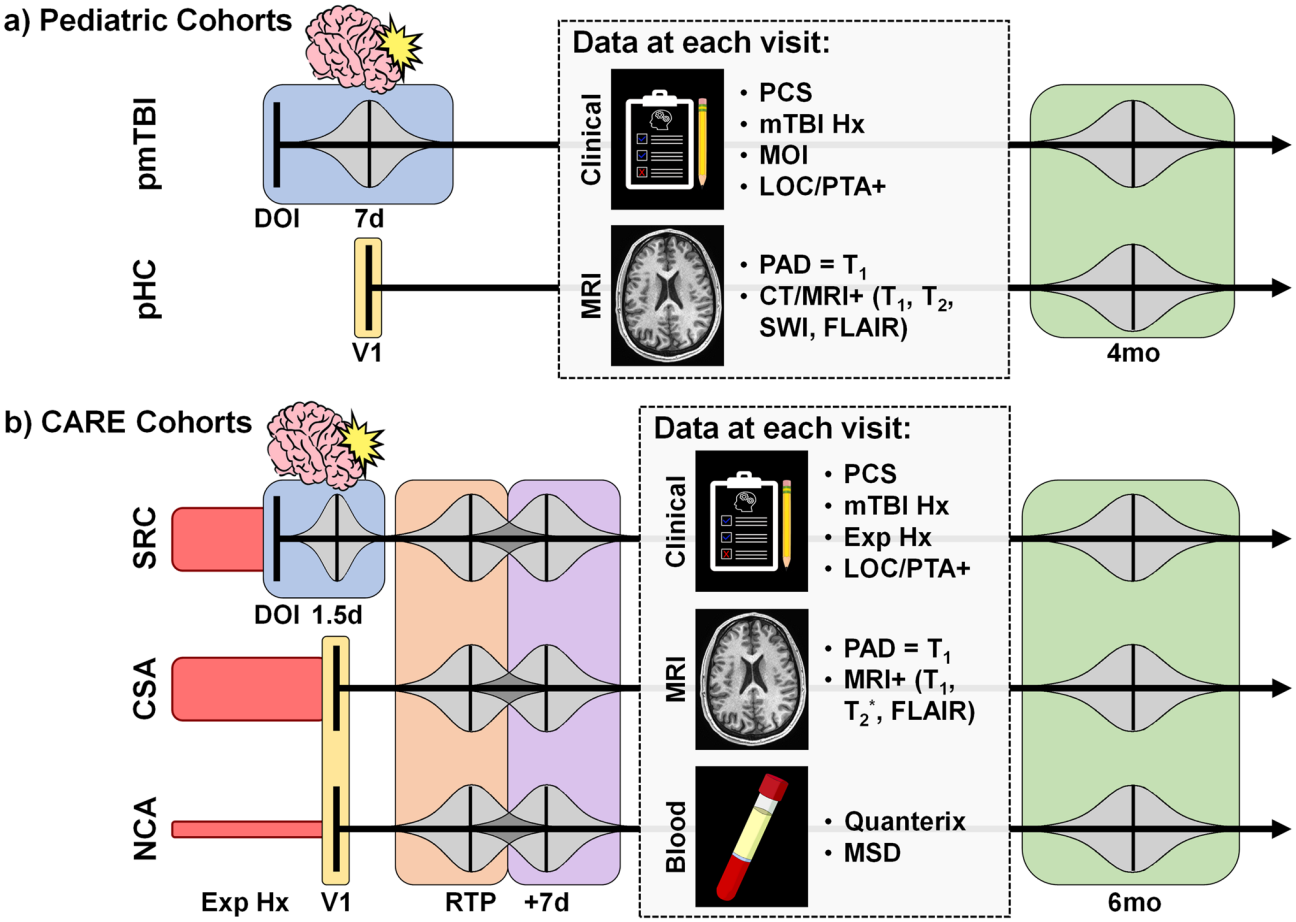
Similarities and differences in the study design for pediatric cohorts (panel **a**) and athlete cohorts from the CARE consortium (panel **b**). Patients with pediatric mild traumatic brain injury (pmTBI) were assessed approximately 7 days (d; blue box) and 4 months (mo; green box) post-injury with multiple magnetic resonance imaging (MRI) sequences [T_1_-weighted (T_1_), T_2_-weighted (T_2_), susceptibility-weighted (SWI) and fluid inversion attenuation recovery (FLAIR) images] and clinical assessments. Age- and sex-matched pediatric healthy controls (pHC) were studied with identical measures at similar temporal intervals (V1 = Visit 1). Computed tomography (CT) scans occurred on the day of injury (DOI) for a subset of pmTBI. Athletes with sport-related concussion (SRC) were assessed approximately 1.5 days (blue box), at return-to-play (RTP; orange box), 7 days after return-to-play (+ 7d; purple) and 6 months post-injury with MRI sequences (T_1_, T_2*_-weighted [T_2*_] and FLAIR images), clinical assessments and blood collection. Age- and sex-matched contact sport athletes (CSA) and non-contact sport athletes (NCA) received identical assessments at similar time-points. Predicted brain age difference (PAD) was determined from T_1_ images for all groups, and all groups were assessed for post-concussive symptom (PCS) burden with age-appropriate scales. Blood data were analyzed on the Quanterix and Meso Scale Delivery (MSD) platforms for CARE data only. Loss of consciousness/post-traumatic amnesia (LOC/PTA), presence of structural imaging findings (CT/MRI +), and mechanism of injury (MOI) were generally higher/more diverse for pmTBI, whereas CARE cohorts (SRC and CSA) had greater exposure history (Exp Hx; denoted by red bars of different heights). Gaussian distributions of different sizes (grey) are used to approximate variability within study visits, as well as the potential for temporally overlapping assessment windows across RTP and + 7d study visits

Pediatric mTBI patients (pmTBI; 8–18 years old) were consecutively recruited from local emergency department and urgent care settings in an ongoing study (initiated in July 2016) and seen at an outpatient facility approximately one week and four months post-injury. Inclusion criteria were a blend of the American Congress of Rehabilitation Medicine (upper injury limit threshold) and the Zurich Concussion in Sport Group (minimal criteria threshold). As such, patients with positive intracranial pathology on CT scans were included in the study. Additional inclusion criteria were Glasgow Coma Score ≥ 13, post-traumatic amnesia (PTA; if present) limited to 24 h, loss of consciousness (LOC; if present) limited to 30 min, alteration in mental status immediately post-injury, or a minimum of two new symptoms post-injury. Injury mechanisms were categorized by cause (motor vehicle crashes, strikes by objects, falls, recreation, etc.) using published criteria [27, 28]. Age- and sex-matched pediatric healthy controls (pHC) were recruited from the local community through flyers and word-of-mouth, and underwent identical assessments at similar time intervals to control for neurodevelopmental confounds (see Fig. 1a).

Exclusion criteria have been previously described [29]. Briefly, participants were excluded based on major neurological, psychiatric, medical or developmental disorders, history of substance abuse/dependence, a previous TBI with greater than 30 min loss of consciousness, contraindications for MRI, or non-English speaking.

A modified version of the 5P risk score was calculated for pediatric participants (see Supplementary Materials). A semi-structured pediatric interview rigorously ascertained previous TBI history. The Post-Concussion Symptom Inventory (PCSI) quantified symptom burden. T_1_-weighted images (1 mm^3^) were acquired on a Siemens 3T scanner using a 32-channel head coil along with other structural sequences (see Supplementary Materials). The T_1_-weighted and additional structural images were reviewed by a board-certified neuroradiologist blinded to participant group [29]. A subset of pmTBI received CT scans as part of standard care.

Brain age data from 5 pmTBI and 1 pHC were eliminated due to being extreme outliers (i.e., more than 3 times inter-quartile range) within their respective cohorts. The final sample (see Supplementary Materials) included 235 pmTBI (98 females; age 14.3 ± 2.9; 7.3 ± 2.3 days post-injury) and 211 pHC (94 females; age 13.9 ± 3.0) for the SA visit. A total of 163 pmTBI (67 females; 124.1 ± 14.7 days between visits) and 193 HC (83 females; 127.3 ± 19.0 days between visits) provided imaging data for the EC visit (see Table 1 for mechanism and severity of injury characteristics).

**Table 1:** Demographics and injury characteristic data for pediatric sample

	~ 7D		~ 4Mo	
	pmTBI (*N* = 235)	pHC (*N* = 211)	pmTBI (*N* = 163)	pHC (*N* = 193)
Age	14.8 (12.4–16.8)	13.9 (11.8–16.3)	14.4 (12.5–16.8)	14.3 (12.2–16.5)
Sex (% F)	41.70%	44.55%	41.10%	43.01%
Tanner	4 (3–4)	4 (2–4)	4 (2–4)	4 (2–4)
Handedness (% R)	91.06%	89.10%	90.80%	89.64%
pmTBI Hx	17.52%	6.16%	20.99%	6.22%
PCSI (% Max)	17 (5.5–38)	3 (0–10)	5 (0.5–17)	4 (1–9)
5P Risk Score	6 (4–7)	3 (2–4)	6 (4–7)	3 (2–4)
Injury characteristics				
MRI/CT +	5.96%	–	7.98%	–
LOC	50.85%	–	48.15%	–
PTA	37.93%	–	35.63%	–
Mechanism of injury				
Struck by object	15.88%	–	16.05%	–
Struck by person	21.46%	–	20.99%	–
Fall	27.04%	–	29.63%	–
MVC	27.04%	–	24.69%	–
Assault	4.29%	–	3.70%	–
Bicycle	3.86%	–	4.32%	–
Other	0.43%	–	0.62%	–
Sport/recreation related	60.09%	–	61.73%	–

Data are formatted as median (interquartile range) based on distribution properties

* ~ 7D* approximately 7 days post-injury, *4Mo* 4 months post-injury, *pHC* pediatric healthy controls, *pmTBI* patients with pediatric mild traumatic brain injury, *F* female, *R* right, *Hx* history, *PCSI* Post-Concussion Symptom Inventory, *MRI/CT* +  positive trauma finding on imaging, *LOC* loss of consciousness, *PTA* post-traumatic amnesia, *MVC* motor vehicle crash

### CARE consortium cohorts

The athlete samples were derived from the Concussion Assessment, Research, and Education (CARE) Consortium and included participants with sport-related concussion (SRC), non-concussed contact sport control athletes with high exposure rates to repetitive head impacts based on primary sport (CSA), and non-concussed non-contact control athletes with lower exposure rates (NCA). Methods for the NCAA-DoD CARE Consortium have been previously described [30]. The current report focuses on athletes completing T_1_-weighted images (1 mm^3^) on 3T (GE or Siemens) scanners with 32-channel head coils (see Supplementary Materials). For the CARE cohort, imaging and clinical data collection were attempted across 4 separate visits. Specifically, athletes with SRC were evaluated at approximately 24–48 h post-injury (1.5 days), following clearance to begin the return-to-play (RTP) progression (asymptomatic), seven days following unrestricted RTP, and 6 months post-injury (Fig. 1b). Concussions were diagnosed by medical staff based on published definitions [31]. CSA and NCA were enrolled at preseason and followed at equivalent imaging and clinical time-points as independent control groups. The T_1_-weighted and additional structural images were reviewed by a board-certified neuroradiologist with a minimum (< 1%) of acute findings on scans [32].

A number of pre-existing medical and psychiatric conditions were present in a small percentage of CARE participants but were not considered exclusionary [30]. Detailed demographic and health history information were collected at baseline. The clinical battery for the current study focuses on measures of symptom severity (Sport Concussion Assessment Tool—3rd Edition symptom checklist; SCAT), repeated at all visits. Exposure was quantified by semi-structured interviews based on participation in contact sports (separate CSA cohort), by concussion history (both CSA and SRC cohorts), and by years of contact sport exposure (both CSA and SRC cohorts).

PAD data from 1 SRC, 1 CSA and 3 NCA were eliminated due to being extreme outliers within their respective cohort. The final sample consisted of 97 SRC (16 females; 20.3 ± 1.1 years old), 82 CSA (17 females; 20.4 ± 1.3 years old), and 87 NCA (17 female; 20.7 ± 1.2 years old) participants. Participant numbers and injury characteristics for each visit are reported in Table 2.

**Table 2:** Demographics and injury characteristic data for CARE sample

	~ 1.5D			RTP			RTP ~ 7			6Mo		
	SRC (*N* = 66)	CSA (*N* = 73)	NCA (*N* = 79)	SRC (*N* = 72)	CSA (*N* = 77)	NCA (*N* = 80)	SRC (*N* = 57)	CSA (*N* = 73)	NCA (*N *= 81)	SRC (*N* = 61)	CSA (*N* = 56)	NCA (*N* = 52)
Age	20.1 ± 1.1	20.3 ± 1.3	20.6 ± 1.3	20.2 ± 1.2	20.3 ± 1.3	20.7 ± 1.2	20.2 ± 1.1	20.4 ± 1.3	20.7 ± 1.2	20.5 ± 1.1	20.5 ± 1.2	21.0 ± 1.2
Sex (% F)	16.67%	21.92%	18.99%	18.06%	22.08%	18.75%	21.05%	21.92%	19.75%	21.31%	23.21%	23.08%
Handedness (% R)	86.36%	84.93%	88.61%	88.89%	85.71%	88.75%	91.23%	86.30%	88.89%	88.52%	91.07%	92.31%
mTBI Hx	63.64%	35.62%	16.46%	55.56%	33.77%	16.25%	54.39%	32.88%	16.05%	59.02%	23.21%	17.31%
SCAT	18 (8–29)	1 (0–3)	1 (0–3)	0 (0–1)	0 (0–2)	0 (0–3.5)	0 (0–0)	0 (0–2)	0 (0–3)	0 (0–1)	0 (0–3.5)	0 (0–2)
Exposure (years)	12 (9–15)	13 (10–15)	2 (0–8)	12.5 (9–15)	13 (10–15)	2 (0–8)	12 (10–15)	13 (10–17)	2 (0–7)	12 (10–16)	13 (10–15)	0 (0–6)

Data are either formatted as mean ± standard deviation or median (interquartile range) based on distribution properties

*SRC* patients with sport-related concussion, *CSA* contact sport control athletes, *NCA* non-contact control athletes; ~ *1.5D* approximately 1.5 days post-injury, *RTP* following return-to-play clearance, *RTP* ~ *7* approximately seven days following unrestricted RTP, 6Mo 6 months post-injury, *F* female, *R* right, *mTBI Hx* mild traumatic brain injury history, *SCAT* Sport Concussion Assessment Tool

### Standard protocol approvals, registrations, and patient consents

The pediatric study was approved by the University of New Mexico School of Medicine Institutional Review Board. All participants provided written informed consent or assent depending on age with parental consent for everyone under 18 years of age. The CARE study was approved by the Medical College of Wisconsin Institutional Review Board and the Department of Defense Human Research Protection Office. All participants provided written informed consent.

### Brain age calculation

**Fig. 2:**
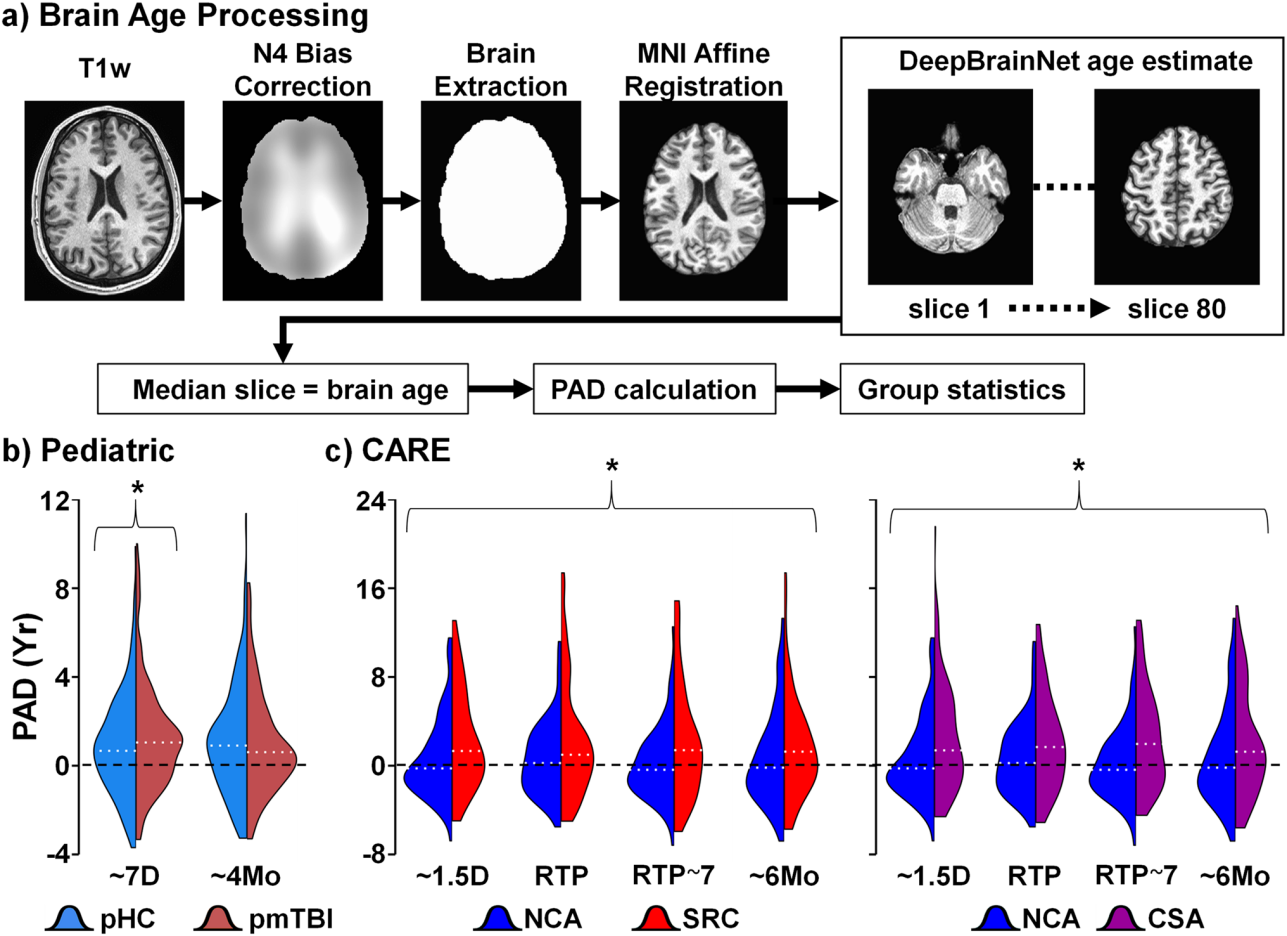
Panel **a** depicts the brain age estimation method. Raw T_1_-weighted data were first preprocessed using N4 bias correction, followed by brain extraction and affine normalization to the Montreal Neurological Institute (MNI) space. The volume was then decomposed into 80 slices, followed by slice-wise estimation of brain age using the published DeepBrainNet algorithm. The median brain age across all 80 slices was selected as the representative brain age statistic to facilitate calculation of predicted brain age difference (PAD: estimated brain age minus chronological age). Panel **b** depicts density plots (relative to maximum density across all relevant timepoints and diagnoses) for predicted age difference data for individuals with pediatric mild traumatic brain injury (pmTBI; maroon kernel) at approximately 7 days (D) and 4 months (Mo) post-injury relative to sex- and age-matched healthy controls (pHC; light blue kernel). Density plots for individuals with sport-related concussion (SRC; red kernel) and non-contact sport athlete controls (NCA; dark blue kernel) from the CARE cohort are presented in panel **c**, as well as for contact sport athlete controls (CSA; purple kernel). Study visits occurred approximately 1.5 days, upon return-to-play (RTP; variable time and dependent on when athlete was asymptomatic), 7 days post return-to-play (RTP ~ 7), and at approximately 6 months post-injury for injured athletes, or at equivalent timepoints for both CARE control groups. For all density plots, a dotted white line indicates the median of the distribution. Both injured and contact sport athletes exhibited increased brain age relative to non-contact athlete controls across all study visits (bracket with asterisk). In contrast, pmTBI participants only exhibited increased brain age at the 7 day visit relative to typically developing children

Analyses were performed in Python (v3.10) with AntsPy (v0.3.4). High-resolution T_1_-weighted images were used as inputs for brain age calculations for all cohorts (Fig. 2a). Brain age was estimated using a deep learning network (DeepBrainNet) architecture [26]. The algorithm was initially trained using a diverse cohort of 11,729 participants aged 3–95 years old from multiple imaging sites. The DeepBrainNet algorithm was purposefully selected for the current study because it is applicable to both the pediatric and collegiate cohorts. Preprocessing steps for T_1_-weighted images included N4 bias field correction, brain extraction, and affine registration to Montreal Neurological Institute (MNI). From the registered volume, 80 axial slices (range 45–125) were extracted and converted to 3-channel RGB format. Conversion to the standard JPEG format [26] resulted in similar findings. Brain age was predicted on a slice-by-slice basis using the trained model with TensorFlow and Keras (2.10). PAD was calculated by subtracting the actual subject age from the median value of all slice-wise predictions.

### Blood collection methods

Blood collection was performed on CARE cohorts only [10]. Non-fasting blood samples were collected by venipuncture (10-mL red-top tube for serum) at all post-injury time points. Samples were centrifuged for 15 min at 1500 RCF within 60 min of collection and aliquoted. Longitudinal samples from the same individual were always run on the same plate, with the three groups randomly distributed across plates. All markers were analyzed in duplicate. Multi-plex technology (Simoa™, Quanterix Corp., Lexington, MA) quantified UCH-L1, total tau, NFL, and GFAP. Samples with concentrations below level of detection or with either intra-assay or inter-assay coefficients of variance above 20% were excluded.

Inflammatory markers (IL-6, IL-10, and TNF-α) were quantified using a Meso Scale Discovery (MSD) QuickPlex SQ 120 instrument and MSD V-PLEX assays from a single lot following manufacturer’s instructions. Inflammatory marker values below the lower limit of detection were replaced by the lower limit of detection and divided by the square root of 2. See Supplemental Table 1 for a more detailed accounting of how many inflammatory markers were replaced in each group. Any duplicates with coefficients of variation greater than 25% were excluded.

### Statistical analyses

Clinical and demographic analyses were conducted with either generalized linear models (GLM; Group effect only) or generalized estimating equations (GEE; group and visit effects), with appropriate distribution (Gaussian, gamma or negative binomial distributions) determined by information criterion results. Brain age analyses were conducted with GEE (group × visit) models with diagnostic status, MRI scanner type, visit and chronological age as covariates as the inclusion of chronological age has been shown to reduce bias. Total intracranial volume estimates were acquired with FreeSurfer (5.3.0) separately for each subject visit in the CARE data, and used as an additional covariate to control for potential differences of head size on brain age estimates. Primary results were similar regardless of whether intracranial volume was included or excluded from the model. Log-transformations were used for all blood biomarker concentration values.

## Results

### Pediatric mTBI cohort

The pmTBI and pHC groups did not differ in terms of handedness, age, self-reported Tanner stage of development, or biological sex (all *p*’s ≥ 0.05; Table 1). Conversely, significant group differences were observed for self-reported previous head injuries (*Χ*^2^ = 13.43, *p* < 0.001; pmTBI = 17.5%, pHC = 6.2%). A total of 14 pmTBI (6.0%) were identified as having a complicated injury based on positive CT or MRI scan results. Approximately one half of pmTBI individuals experienced either LOC (~ 50.9%) and/or PTA (~ 37.9%). The majority of injuries in the pmTBI sample (60.1%; Table 1) resulted from sport or recreation-related causes.

As expected, results for self-reported PCS severity indicated a significant group × visit interaction (Wald-*χ*^*2*^ = 29.80; *p* < 0.001), with relatively higher PCS severity for pmTBI relative to HC at the SA (Wald-*χ*^*2*^ = 105.08; *p* < 0.001) compared to EC (Wald-*χ*^*2*^ = 7.19; *p* = 0.007) visit indicative of incomplete recovery in a minority of participants. There were no differences in demographics or PCS severity for returning versus non-returning pmTBI or pHC participants (all *p*’s > 0.05).

Results (Fig. 2b) from a 2 × 2 (group × visit) GEE for PAD with chronological age and scanner type as nuisance covariates indicated a significant group × visit interaction (Wald-*χ*^*2*^ = 9.29; *p* = 0.002). Follow-up GLM tests indicated that PAD was significantly increased by approximately 6 months at the SA visit (Wald-*χ*^*2*^ = 4.45; *p* = 0.035; Cohen’s *d* = 0.17) for pmTBI (PAD marginal mean = 1.62 ± 2.85 years) relative to HC (PAD marginal mean = 1.16 ± 2.73 years), whereas there were no significant group differences at EC visit (Wald-*χ*^*2*^ = 0.22; *p* = 0.636; Cohen’s *d* = -0.04).

The next set of analyses examined the relationship between clinical gold-standards (5P risk score, PCS load), indices of injury severity (LOC, PTA, complicated pmTBI status), history of concussion, and mechanism of injury (sport-related injury versus non-sport related injuries) with PAD at the sub-acute visit for pmTBI only. Results indicated that the 5P risk score was negatively associated (*β* = − 0.183; *p* = 0.037) with PAD, while PTA severity was positively associated with PAD (*β* = 0.384; *p* = 0.003). History of concussions, complicated pmTBI and mechanism of injury did not account for any unique variance.

### CARE consortium cohorts

CARE cohorts did not differ in terms of handedness, age or biological sex (all *p*’s > 0.05; see Table 2). As expected, there were significant group differences in terms of previous self-reported history of concussion across the three groups (*Χ*^2^ = 34.39, *p* < 0.001; SRC = 63.6%, CSA = 35.6%, NCA = 16.5%). In terms of symptom load from the SCAT, a 3 × 4 GEE indicated significant group × visit interaction (Wald-*χ*^2^ = 133.58; *p* < 0.001), with a large increase in symptom burden for the SRC group at the 1.5 day visit (Wald-*χ*^2^ = 141.70) relative to both CSA (*p* < 0.001) and NCA (*p* < 0.001). SRC symptom scores were significantly lower than CSA at all three remaining visits (all *p*’s ≤ 0.009), and lower than NCA at both the asymptomatic (*p* < 0.001) and 7-day post-RTP visits (*p* < 0.001). In contrast to the pmTBI cohort, there were minimal numbers of individuals with either LOC (~ 5.9%) or PTA (~ 12.1%) within the SRC cohort.

There were no differences in handedness or biological sex between returning and non-returning participants (*p*’s > 0.05). However, returning SRC and CSA were younger (uncorrected *p*’s < 0.05) than non-returning counterparts from the same group. Additionally, returning CSA reported higher symptom burden relative to non-returning CSA (uncorrected *p* = 0.001). No differences in symptom burden were observed for returning versus non-returning SRC or NCA (*p*’s > 0.05).

Results (Fig. 2c) from a 3 × 4 (group × visit) GEE for PAD differences with chronological age, scanner type, and estimated intracranial volume as nuisance covariates resulted in a significant main effect for Group (Wald-*χ*^2^ = 18.14; *p* < 0.001). Follow-up pairwise GLM tests indicated that PAD was significantly increased for the SRC (PAD marginal mean = 2.53 ± 4.62 years) relative to NCA (PAD marginal mean = 0.23 ± 3.93 years) group by approximately 2.3 years (Wald-*χ*^2^ = 12.71; *p* < 0.001), but not for the SRC relative to CSA (PAD marginal mean = 2.42 ± 4.66 years) group (*p* > 0.05). Similarly, CSA also exhibited increased PAD relative to NCA by approximately 2.2 years (Wald-*χ*^2^ = 10.84; *p* = 0.001).

Although the group × visit interaction was not significant for CARE data, pairwise tests were conducted at each visit as secondary, unplanned analyses given results from the pediatric cohort and the potential for low power to detect interactions across the 4 study visits. Results indicated that both SRC (*p* range = 0.007–0.031; *d* range = 0.34–0.47) and CSA (*p* range = 0.0003–0.018; *d* range = 0.35–0.54) exhibited significant differences relative to NCA at all visits with the exception of 6 months post-injury (both *p*’s > 0.05; SRC-NCA *d* = 0.18, CSA-NCA *d* = 0.24). In addition to the reduced effect sizes, sample sizes for both control groups were also smaller at the 6-month follow-up (see Table 2).

The relationship between PAD and PCS load, indices of injury severity (LOC, PTA), past history of concussion, and years of exposure to contact sports was examined in the SRC cohort with chronological age and scanner type as nuisance variables. Data were included for the first three visits (1.5 days, asymptomatic, and 7 days post-RTP) based on PAD findings, with visit modeled as an additional factor. Results for this analysis were null (*p* > 0.05). After eliminating the acute injury severity variables, a similar analysis was repeated in CSA athletes. Results were also null for history of concussion and years of exposure to contact sports (*p* > 0.05).

### CARE blood-based biomarkers

Blood-based biomarker results have been previously reported in a larger CARE cohort [10]. The current investigation included only a sub-sample (~ 20% of previously published data) of participants with concurrent imaging data. Blood-based biomarker analyses were also limited to the first 3 visits (1.5 days, asymptomatic and 7 days post-RTP) used in the current study. Bonferroni correction was completed separately for neural (0.05/4 = 0.0125) relative to inflammatory (0.05/3 = 0.0167) biomarkers.

Results (Table 3) from the 3 × 3 (group × visit) GEEs indicated no significant group differences or interactions for IL-10, TNF-α, GFAP or NFL across the first three study visits (all *p*’s > 0.05). A main effect of group was significant for IL-6 (Wald-*χ*^2^ = 8.23; *p* = 0.016), with both SRC (*p* = 0.011) and CSA (*p* = 0.020) demonstrating increased IL-6 levels relative to NCA. The group × visit interaction was significant for both total tau (Wald-*χ*^2^ = 15.85; *p* = 0.003) and UCH-L1 (Wald-*χ*^2^ = 13.63; *p* = 0.009) following Bonferroni correction. Follow-up simple effects tests for total tau indicated that the group effect was significant only at the ~ 1.5 day visit, with SRC exhibiting significantly lower levels relative to CSA and NCA (both p’s < 0.05). In contrast, there were no significant simple effects for UCH-L1 during post-hoc interaction tests.

**Table 3:** Log-transformed concentration values for blood-based biomarkers from the CARE sample

	~ 1.5D			RTP			RTP ~ 7		
	SRC	CSA	NCA	SRC	CSA	NCA	SRC	CSA	NCA
TNF-α	− 0.17 ± 0.48	− 0.22 ± 0.40	− 0.18 ± 0.36	− 0.13 ± 0.49	− 0.23 ± 0.42	− 0.22 ± 0.42	− 0.12 ± 0.43	− 0.19 ± 0.44	− 0.18 ± 0.40
IL-6*	− 1.11 ± 1.10	− 1.19 ± 0.96	− 1.44 ± 0.88	− 1.12 ± 0.97	− 1.34 ± 0.87	− 1.37 ± 0.97	− 1.09 ± 1.08	− 1.18 ± 0.94	− 1.59 ± 0.83
IL-10	− 1.35 ± 1.19	− 1.53 ± 1.10	− 1.43 ± 0.86	− 1.34 ± 1.11	− 1.63 ± 0.85	− 1.47 ± 0.75	− 1.28 ± 1.05	− 1.52 ± 1.02	− 1.58 ± 0.62
NFL	1.71 ± 0.44	1.77 ± 0.51	1.78 ± 0.38	1.76 ± 0.54	1.83 ± 0.45	1.80 ± 0.39	1.68 ± 0.41	1.83 ± 0.51	1.84 ± 0.41
UCH-L1҂	2.35 ± 0.99	2.46 ± 1.04	2.44 ± 0.82	2.21 ± 0.93	2.47 ± 1.03	2.50 ± 0.88	2.63 ± 0.98	2.36 ± 0.86	2.34 ± 1.15
GFAP	4.36 ± 0.74	4.16 ± 0.43	4.20 ± 0.52	4.17 ± 0.46	4.18 ± 0.44	4.17 ± 0.51	4.16 ± 0.35	4.12 ± 0.43	4.25 ± 0.49
T-tau҂	− 0.52 ± 0.69	− 0.28 ± 0.79	− 0.29 ± 0.65	− 0.15 ± 0.77	− 0.23 ± 0.80	− 0.30 ± 0.70	0.00 ± 0.55	− 0.20 ± 0.62	− 0.20 ± 0.63

Tumor necrosis factor alpha (TNF-α); interleukins 6 (IL-6) and 10 (IL-10); neurofilament light chain (NFL); ubiquitin C-terminal hydrolase L1 (UCH-L1); glial fibrillary acidic protein (GFAP), total tau (T-tau). All values are displayed as a log-transformed concentration (unit = log of picogram/milliliter pg/mL) and formatted as mean ± standard deviation. Significant group × visit interactions (denoted with a cross) existed for UCH-L1 (non-significant follow-ups) and T-tau (different only at 1.5D), whereas an asterisk is used to denote significant main effect of group (IL-6)

*SRC* athletes with sport-related concussion, *CSA* contact sport control athletes, *NCA* non-contact control athletes; ~ *1.5D* approximately 1.5 days post-injury, *RTP* following return-to-play clearance, *RTP* ~ *7* approximately seven days following unrestricted RTP

The final series of analyses examined associations between blood-based biomarkers and PAD separately for the SRC and CSA cohorts across the first three visits. Results from the SRC cohort indicated a negative association between IL-10 and PAD (*β* = − 1.145; *p* = 0.014), as well as a trend positive association between NFL and PAD (*β* = 1.646; *p* = 0.095). In contrast, only the visit variable was significant (Wald-*χ*^2^ = 6.50; *p* = 0.039) within the CSA cohort.

## Discussion

Neuropathological change post-TBI remains an actively debated topic, with recent systematic reviews suggesting stronger evidence for atrophy in msTBI relative to mTBI, as well as for changes that occur during more chronic relative to sub-acute stages of mTBI [1, 7]. The current study observed increased brain age during the acute (CARE cohort) and sub-acute (pediatric and CARE cohorts) stages of mTBI in two relatively young, replicated samples, but with potentially varying recovery trajectories (i.e., pediatric recovering more rapidly than athletes). The pediatric and athlete samples also differed across multiple traditional markers of injury severity (presence/absence of lesions on structural imaging, percentage with loss of consciousness/post-traumatic amnesia, mechanisms of injury) and exposure history. Mixed evidence on the effects of repetitive head impact exposure on brain age were observed. Specifically, non-concussed contact sport athletes with high exposure to repetitive hits also demonstrated significantly increased brain age relative to non-contact sport controls. However, there were no relationships between brain age and concussion history (pediatric and CARE cohorts) or with years of exposure to contact sports (CARE cohort).

Previous studies in adult or geriatric mTBI cohorts provide conflicting reports of chronic increases in brain age post-injury [22–24] as well as null findings [15]. Although both pediatric and acutely concussed athlete samples exhibited increased brain age during the sub-acute injury phase, only the pediatric cohort demonstrated evidence of recovery multiple months post-injury. Similar to previous findings in adult samples [22, 23], the collegiate athlete sample demonstrated stronger evidence for chronically increased brain age, although these findings may have been limited by power and/or influenced by exposure to repetitive injury effects. Although increased brain age is most typically attributed to neurodegeneration [14, 33], these two concepts are not synonymous. Specifically, post-traumatic pathologies with potentially reversible causes (vasogenic or cytotoxic edema, inflammatory processes; see discussion below) could also contribute to calculated and temporary differences in brain age due to widening of the sulci [34]. Similarly, even short-term changes in environmental factors such as diet can potentially affect brain structure post-injury [35]. Finally, it is critical to note that the clinical and functional significance of increased brain age remains unclear in both TBI and other neuropsychiatric populations [33].

It is also notable that both cohorts were likely undergoing active neurodevelopmental changes within and across the study intervals, which further complicates the interpretation of brain age estimates and the potential superimposition of trauma-related pathology. Specifically, cortical thinning represents the prototypical and most pronounced morphological neurodevelopmental trajectory during adolescence [36], and occurs even over 4–6 month intervals [37]. In this context, increased brain age may represent an altered neurodevelopmental trajectory rather than neurodegeneration as is commonly prescribed in other neuropsychiatric and geriatric samples [14]. Importantly, large sample sizes across the entire lifespan were used to train the DeepBrainNet algorithm utilized in the current study [26], which should make it more robust to these developmental effects relative to other brain age estimate methods. However, the DeepBrainNet algorithm tended to over-estimate brain age for even the pediatric control samples in the current study by approximately 1.16 years, which may be indicative of either algorithm bias or sample bias.

“Exposure history” has been variably quantified in past studies by examining individuals who are active contact sport athletes, by quantifying the frequency of previous concussions, by quantifying the duration of exposure (e.g., years of play), or by quantifying age of first exposure to repetitive head impacts without concussion [5, 38]. Similar to the acutely concussed group, contact sport athletes also exhibited approximately the same magnitude of increased brain age relative to the non-contact control group, as well as increased peripheral markers of inflammation (IL-6). This suggests that findings of increased brain age or inflammation are not specific to an acute concussive event and may be secondary to the cumulative effects of repetitive head impact exposure. However, neither frequency of previous concussions (pediatric and CARE cohorts) nor years of contact sport exposure (CARE concussed and contact control cohort) was associated with increased brain age. Previous studies have also found minimal evidence (less than 1%) of radiological findings on structural images in the CARE cohort [32].

Repetitive injury has been associated with macrostructural changes (e.g., cavum septum pellucidum with septal fenestrations, ventriculomegaly, atrophy) in very chronic mTBI [3, 39–41]. However, exposure effects are mixed in more acute or younger samples [42], including within the CARE consortium [43]. Some previous studies have reported morphological changes in brain microstructure and volumetrics after a single season of play [44, 45], but these findings have not been replicated in other studies. It is also important to note that the effects of years of exposure and concussion history were independently assessed within the acutely concussion and contact sport control group rather compared between these groups and non-contact athletes. This approach reduces circular analyses (also known as “double-dipping” and “voodoo correlations”) given the existing differences between concussed/contact athletes and non-contact controls on both exposure history and brain age [46, 47].

It would be overly-speculative to suggest that repetitive injury may lead to neurodegenerative changes (chronic traumatic encephalopathy or others) as indexed by brain age based on the current results alone. Although we controlled for potential differences in head size, systematic differences in general physiology and environment could also contribute to current brain age findings [18]. Longitudinal studies in large athlete samples over longer durations are ultimately required to determine whether these differences in brain age persist and/or increase over time, which is the necessary study design for concluding that brain age differences are truly degenerative. Previous brain age studies in adult mTBI have either excluded individuals with a history of repetitive mTBI [23] or have also reported a null association between concussion history and brain age [24]. Thus, it is likely that neither exposure to concussion nor repetitive head impacts represent singular or combined causes for increased brain age, and that a matrix of factors contribute to increased brain age among athletes. Quantifying exposure in terms of years of participation or past number of head injuries (currently the most widely used conventions) will therefore likely remain a highly controversial topic until better precision is achieved [48].

There was no association between brain age and the magnitude of post-concussive symptoms for either sample. Post-concussive symptoms exhibit relatively poor psychometric properties [49] and resolution is dependent on both age (adults resolve faster than children) [50] and sample (sport-related concussions resolve faster than emergency room cohorts) [51]. In contrast to brain age findings, the SRC sample reported a rapid and pronounced recovery based on self-report (i.e., symptoms lower than control groups following return-to-play protocol) whereas a minority of the pmTBI cohort remained symptomatic at 4 months post-injury. Thus, it may be particularly challenging to develop biological markers of persistent PCS, even when pathology is captured by a single scalar value such as brain age. In contrast, PTA duration was associated with brain age in our pediatric sample, as has been previously observed in msTBI studies with various injury severity characteristics (e.g., duration of PTA, Glasgow Coma Scale) [21] including cognitive dysfunction [15, 21]. Current and previous data from the entire TBI spectrum collectively suggest a potential dose-dependent relationship between injury severity and changes in brain age [15]. In contrast, the 5P risk score, which amalgamates demographic (e.g., age and sex), concussion injury history, certain PCS and balance into a single score, was negatively associated with brain age in pmTBI. This association will therefore both require independent replication and an examination of individual components to further clarify the potential relationships with brain age.

Blood-based biomarker differences for SRC were only observed for total tau, which was decreased relative to both contact and non-contract controls at the 1.5 day assessment. Decreased tau was previously reported in the full CARE sample at this timepoint, although it was elevated more immediately following concussion [10]. Furthermore, previous independent studies have reported decreased [52], increased [53, 54] and null [55] findings for tau across the acute phases of sport-related concussion, which could be partially explained by differences in post-injury collection times across studies. More recent studies suggest that there is also minimal correspondence between serum levels of total tau and tau derived from cerebral spinal fluid [56], questioning the specificity of this marker. Finally, other studies suggest that changes in tau levels may occur after rigorous exercise [57], with mandatory rest protocols post-concussion therefore potentially also affecting total tau levels relative to other more active athletes. The minimal sensitivity of GFAP, UCH-L1 and NFL in the current sample relative to the full CARE cohort [10, 11] likely results from both reduced power (i.e., inclusion to current study predicated on presence of imaging data) and exclusion of hyperacute (< 24 h) blood samples. Specifically, blood biomarkers typically exhibit the largest signal in the first few hours post-mTBI, especially for GFAP and UCH-L1 [10, 11]. Although elevated exosome concentrations of p-tau, NFL, IL-6, TNF-α, and GFAP have also been reported decades post-TBI in a mixed severity military sample [58], it is not clear if these findings were specific to TBI or other neurodegenerative factors.

Previous studies of msTBI have observed a positive relationship between NFL and increased brain age and/or volume loss across independent samples of msTBI [12, 19, 20]. In contrast, the association between NFL and brain age following SRC was only at a trend level in the current study. Collectively, current and previous results suggest that NFL may represent a blood-based marker of brain pathology and advanced age in a dose-dependent fashion, but that larger sample sizes are required to observe this effect. IL-6 was elevated in both concussed and contact-sport control cohorts. Senescent cells release pro-inflammatory signals which have been associated with neurodegeneration in both typical and atypical aging (inflammageing), and affect the blood–brain barrier [59, 60]. There was not a linear relationship between IL-6 and increased brain age estimates, but the anti-inflammatory cytokine IL-10 was inversely related to brain age in concussed athletes. Although preliminary, these findings collectively suggest a possible cycle of pro-inflammation followed by an increased anti-inflammatory response (IL-10) which may reduce blood–brain barrier dysfunction and subsequent edema, resulting in narrower sulci and more typical brain age in some individuals [61]. However, additional studies are needed to further examine relationships between inflammatory markers and changes in brain age, especially for more chronic TBI samples.

Strengths of the current study include large sample sizes with age- and sex-matched control groups, reducing the likelihood of cohort biases that have been shown to confound brain age estimations [14]. However, there are multiple other environmental and individual host factors that could potentially affect brain age [18] that were not considered in the current design. Second, although findings of increased brain age during the sub-acute injury stage were replicated across independent cohorts (minimum *N* > 50) across the spectrum of mTBI, sample sizes may not have been sufficient to detect smaller effects (e.g., association between NFL and brain age) or interactions (i.e., reduced differences in brain age at 6 months in CARE cohort) or to fully examine other effects (biological sex, stage of puberty). Third, the pediatric study utilized a typically developing rather than orthopedically injured control group. Orthopedic injury groups may better control for non-specific effects of trauma (e.g., pain, disruptions to daily life) on clinical biomarkers as well as some imaging biomarkers [62]. Fourth, the current study utilized only a single modality (structural images) to assess brain age based on the DeepBrainNet algorithm rather than using diffusion or multiple imaging modalities [22].

Finally, an inherent tradeoff of the brain age method is the simplicity of clinical interpretation (i.e., single scalar value) relative to the potential sensitivity to more focal patterns of atrophy. TBI preferentially affects the lateral and basal frontotemporal cortices due to skull morphology, as well as the cerebellum, deep grey (hippocampus, limbic circuitry) and deep white (corpus callosum and brainstem) matter due to the accumulation of shear stresses during inertial loading in these regions [7, 63]. Thus, it is possible that more spatially focal metrics (vertex-wise measures of cortical thinning or certain sub-cortical structures such as the hippocampi) may be more sensitive to effects associated with acute injury or exposure relative to the more general brain age index [8, 9, 64, 65].

In summary, brain age estimates are increasingly applied to the spectrum of TBI during acute to chronic injury phases [15, 21–24]. The current study examined alterations in brain age across the spectrum of mTBI [25], partially replicating findings across multiple cohorts, and utilized rigorous clinical methods (i.e., semi-structured interviews) to quantify exposure history (contact sport participation and concussion history). Current and previous [22, 23] results suggest that the chronicity of brain age differences may be mediated by age at injury (adults > children), potentially as a result of increased neuroplasticity, neurodevelopmental factors or fewer comorbidities in youth [14, 33]. Prospective studies with larger sample sizes in replicated mTBI cohorts are ultimately needed to better address these relationships, including any putative relationships with blood-based biomarkers and changes in brain age as a result of repetitive head impacts.

### Supplementary Information

Below is the link to the electronic supplementary material.

Supplementary file1 (DOCX 28 KB)

## Data Availability

The pediatric data that support the findings of this study will be openly available in FITBIR at fitbir.nih.gov, reference number FITBIR-STUDY0000339 at the conclusion of this study. Data from the CARE cohort are available at the same location under reference number FITBIR-STUDY0000310.
