# Supplementary material for: Increased brain age and relationships with blood-based biomarkers following concussion in younger populations
Source: J Neurol. 2023 Aug 18;270(12):5835–48. doi: 10.1007/s00415-023-11931-8 (PMC10632216; doi:10.1007/s00415-023-11931-8)
Supplement: Supplementary file 1 — Supplementary file1 (DOCX 28 KB) [file 415_2023_11931_MOESM1_ESM.docx]

*Supplementary Materials for “Increased Brain Age and Relationships with Blood-based Biomarkers Following Concussion in Younger Populations” in Journal of Neurology*

Andrew R. Mayer PhD^1,2,3^*, Timothy B. Meier PhD^4,5,6^, Josef M. Ling BA^1^, Andrew B. Dodd MS^1^, Benjamin L. Brett PhD^4,7^, Cidney R. Robertson-Benta BS^1^, Daniel L. Huber MPH^4^, Harm J. Van der Horn MD PhD^1^, Steven P. Broglio PhD^8^, Michael A. McCrea PhD^4,7^, and Thomas McAllister MD^9^.

^1^The Mind Research Network/Lovelace Biomedical and Environmental Research Institute, Albuquerque, New Mexico, USA

^2^Neurology and Psychiatry Departments, University of New Mexico School of Medicine, Albuquerque, New Mexico, USA

^3^Department of Psychology, University of New Mexico, Albuquerque, New Mexico, USA

^4^Department of Neurosurgery, Medical College of Wisconsin, Milwaukee, Wisconsin, USA

^5^Department of Biomedical Engineering, Medical College of Wisconsin, Milwaukee, Wisconsin, USA

^6^Department of Cell Biology, Neurobiology and Anatomy, Medical College of Wisconsin, Milwaukee, Wisconsin, USA

^7^Department of Neurology, Medical College of Wisconsin, Milwaukee, Wisconsin, USA

^8^Michigan Concussion Center, University of Michigan, Ann Arbor, Michigan, USA

^9^Department of Psychiatry, Indiana University School of Medicine, Bloomington, Indiana, USA

*Corresponding Author and Reprint Requests:

Andrew R. Mayer, 1101 Yale Blvd. NE, Albuquerque, NM 87106

Email address: [amayer@mrn.org](mailto:amayer@mrn.org)

Phone number: 505-272-0769

Fax number: 505-272-8002

ORCID: 0000-0003-2396-5609

*Pediatric Cohort Clinical Data*

The final sample included 236 pmTBI (98 females; age 14.3±2.8; 7.3±2.3 days post-injury) and 211 pHC (94 females; age 13.9±3.0) for the SA visit. Prior to exclusions made based on data quality or study specific criteria, a total of 193 pmTBI (81.2% retention) and 197 pHC (93.4% retention) returned for a follow-up visit. Due to a planned equipment upgrade to the scanner, 7 pmTBI returned for clinical data collection only. Of those remaining, 22 pmTBI and 4 pHC were eliminated due to new study exclusion criteria (braces, pregnancy, drug use, etc.) or image quality, leaving a total of 164 pmTBI (67 females; 124.1±14.7 days between visits) and 193 HC (83 females; 127.3±19.0 days between visits) for the EC visit.

Categorical representations of loss of consciousness (LOC) and post-traumatic amnesia (PTA) were used due to their superior psychometric properties relative to a continuous measurement [1]. The 5P risk score [2] was calculated with minor modifications based on available clinical data. Standard calculations included age, sex, prior concussion history and symptom duration, and physician-diagnosed migraine history, as well as headaches, sensitivity to noise and fatigue on the Post-Concussion Symptom Inventory (PCSI) parent form. Modifications in the current study included use of a tandem gait task rather than the recommended mBESS tandem stance, and the "answers questions more slowly" from the PCSI parent form rather than from the Acute Concussion Evaluation. A severity score was calculated for each factor and then summed for a total risk score using recommended criteria [2].

The PCSI was modified in the following ways with author permission: 1) the version of the PCSI for 13-18 year old was also utilized for 12 year old participants, and 2) all references to an injury were removed from both questionnaires and instructions to avoid bias in HC. All summary scores from the PCSI were normalized into percentage values given that different scales were administered to older (ages 12-18) and younger children (ages 8-11).

*Pediatric Cohort Imaging Parameters*

All participants were scanned on a 3T TrioTim or Prisma Fit system (Siemens; Erlangen, Germany) with a 32-channel head coil. A high resolution 5-echo Magnetization Prepared Rapid Acquisition Gradient Echo (MPRAGE) T_1_–weighted [repetition time (TR)=2530 ms; echo times (TE)=1.64, 3.50, 5.36, 7.22, 9.08 ms; inversion time (TI)=1200 ms; flip angle=7°; number of excitations (NEX)=1; slice thickness=1 mm; field of view (FOV)=256 mm; matrix size=256 x 256; isotropic voxels=1 mm^3^] was collected in addition to other structural scans **(T_2_-weighted images, susceptibility-weighted images and fluid inversion attenuation recovery images)**. Foam padding was used to limit head motion on all scan sequences.

*CARE Cohort Imaging Parameters*

High-resolution T_1_-weighted images (1 mm^3^) were acquired on 3T MRI scanners at each site. Specifically, a 3D magnetization-prepared rapid gradient-echo sequence was collected on Siemens MAGNETOM Prisma (32-channel head coil) and MAGNETOM Trio (32- or 12-channel head coils) scanners at UNC and UCLA with the following parameters: TR/TE/TI = 2,300/2.98/900 ms, flip angle = 9°, FOV = 256 mm, matrix = 256 × 256, 176 slices. A 3D Brain Volume (BRAVO) sequence was collected on a General Electric Discovery MR750 scanner with the following parameters: TR = 6.62 to 6.652, TE = 2.91 to 2.928, TI = 450 ms, flip angle = 12°, FOV = 256 mm, matrix = 256 × 256, 164 slices. **Other structural scans** **(T_2_-weighted images, T_2*_-weighted images, and fluid inversion attenuation recovery images) were also collected and reviewed by a neuroradiologist.**

*Brain Age Calculation*

The original brain age methodology Bashyam and colleagues converted slices to JPEG files rather than a 3-channel RGB format which results in data loss. However, primary findings from both the pediatric and CARE cohorts replicated regardless of whether the JPEG or RGB methodology was employed.
 Brain age analyses were conducted with GEE (Group×Visit) models with diagnostic status, MRI scanner type, visit and chronological age as covariates as the inclusion of chronological age has been shown to reduce bias [3].

**References**

1. Hergert DC, Sicard V, Stephenson DD, Pabbathi RS, Robertson-Benta CR, Dodd AB, Bedrick EJ, Gioia GA, Meier TB, Shaff NA, Quinn DK, Campbell RA, Phillips JP, Vakhtin AA, Sapien RE, Mayer AR (2022) Test-Retest Reliability of a Semi-Structured Interview to Aid in Pediatric Traumatic Brain Injury Diagnosis. J IntNeuropsycholSoc 28:687-699

2. Zemek R, Barrowman N, Freedman SB, Gravel J, Gagnon I, McGahern C, Aglipay M, Sangha G, Boutis K, Beer D, Craig W, Burns E, Farion KJ, Mikrogianakis A, Barlow K, Dubrovsky AS, Meeuwisse W, Gioia G, Meehan WP, III, Beauchamp MH, Kamil Y, Grool AM, Hoshizaki B, Anderson P, Brooks BL, Yeates KO, Vassilyadi M, Klassen T, Keightley M, Richer L, DeMatteo C, Osmond MH (2016) Clinical risk score for persistent postconcussion symptoms among children with acute concussion in the ED. JAMA 315:1014-1025

3. Smith SM, Vidaurre D, Alfaro-Almagro F, Nichols TE, Miller KL (2019) Estimation of brain age delta from brain imaging. Neuroimage 200:528-539

Supplemental Table 1: Percentage of participants with either CV > 25 or one duplicate below LLOD and CV > 25 for each of the inflammatory markers (IL-6, IL-10, and TNF-α)

|  | ~1.5D | | | RTP | | | RTP~7 | | | 6Mo | | |
| --- | --- | --- | --- | --- | --- | --- | --- | --- | --- | --- | --- | --- |
|  | SRC (N=63) | CSA  (N=70) | NCA  (N=74) | SRC  (N=69) | CSA  (N=71) | NCA  (N=76) | SRC (N=53) | CSA  (N=70) | NCA  (N=76) | SRC  (N=57) | CSA  (N=52) | NCA  (N=48) |
| IL-6 | 7.9% | 14.3% | 20.3% | 5.8% | 22.5% | 15.8% | 5.7% | 12.9% | 23.7% | 8.8% | 17.3% | 10.4% |
| IL-10 | 1.6% | 2.9% | 2.7% | 1.4% | 2.8% | 2.6% | 0% | 1.4% | 0% | 7.0% | 1.9% | 2.1% |
| TNF-α | 1.6% | 2.9% | 4.1% | 1.4% | 1.4% | 0% | 0% | 1.4% | 2.6% | 3.5% | 0% | 0% |

Notes: SRC=patients with sport-related concussion; CSA=contact sport control athletes; NCA=non-contact control athletes; ~1.5D=approximately 1.5 days post-injury; RTP=following return-to-play clearance; RTP~7=approximately seven days following unrestricted RTP; 6Mo=6 months post-injury.
